# Optic Radiation Tractography in Pediatric Brain Surgery Applications: A Reliability and Agreement Assessment of the Tractography Method

**DOI:** 10.3389/fnins.2019.01254

**Published:** 2019-11-20

**Authors:** Joseph Yuan-Mou Yang, Richard Beare, Michelle Hao Wu, Sarah M. Barton, Charles B. Malpas, Chun-Hung Yeh, A. Simon Harvey, Vicki Anderson, Wirginia J. Maixner, Marc Seal

**Affiliations:** ^1^Department of Neurosurgery, The Royal Children’s Hospital, Melbourne, VIC, Australia; ^2^Developmental Imaging, Murdoch Children’s Research Institute, Melbourne, VIC, Australia; ^3^Neuroscience Research, Murdoch Children’s Research Institute, Melbourne, VIC, Australia; ^4^Department of Paediatrics, The University of Melbourne, Melbourne, VIC, Australia; ^5^Department of Medicine, Monash University, Melbourne, VIC, Australia; ^6^Medical Imaging, The Royal Children’s Hospital, Melbourne, VIC, Australia; ^7^Department of Neurology, The Royal Children’s Hospital, Melbourne, VIC, Australia; ^8^Clinical Outcomes Research Unit, Department of Medicine, Royal Melbourne Hospital, The University of Melbourne, Melbourne, VIC, Australia; ^9^Melbourne School of Psychological Sciences, The University of Melbourne, Melbourne, VIC, Australia; ^10^The Florey Institute of Neuroscience and Mental Health, Melbourne, VIC, Australia; ^11^Brain and Mind, Murdoch Children’s Research Institute, Melbourne, VIC, Australia; ^12^Department of Psychology, The Royal Children’s Hospital, Melbourne, VIC, Australia

**Keywords:** diffusion magnetic resonance imaging, neurosurgery, tractography, Meyer’s loop, optic radiation, visual field deficits

## Abstract

**Background:**

Optic radiation (OR) tractography may help predict and reduce post-neurosurgical visual field deficits. OR tractography methods currently lack pediatric and surgical focus.

**Purpose:**

We propose a clinically feasible OR tractography strategy in a pediatric neurosurgery setting and examine its intra-rater and inter-rater reliability/agreements.

**Methods:**

Preoperative and intraoperative MRI data were obtained from six epilepsy and two brain tumor patients on 3 Tesla MRI scanners. Four raters with different clinical experience followed the proposed strategy to perform probabilistic OR tractography with manually drawing anatomical landmarks to reconstruct the OR pathway, based on fiber orientation distributions estimated from high angular resolution diffusion imaging data. Intra- and inter-rater reliabilities/agreements of tractography results were assessed using intraclass correlation coefficient (ICC) and dice similarity coefficient (DSC) across various tractography and OR morphological metrics, including the lateral geniculate body positions, tract volumes, and Meyer’s loop position from temporal anatomical landmarks.

**Results:**

Good to excellent intra- and inter-rater reproducibility was demonstrated for the majority of OR reconstructions (ICC = 0.70–0.99; DSC = 0.84–0.89). ICC was higher for non-lesional (0.82–0.99) than lesional OR (0.70–0.99). The non-lesional OR’s mean volume was 22.66 cm^3^; the mean Meyer’s loop position was 29.4 mm from the temporal pole, 5.89 mm behind of and 10.26 mm in front of the temporal ventricular horn. The greatest variations (± 1.00–3.00 mm) were observed near pathology, at the tract edges or at cortical endpoints. The OR tractography were used to assist surgical planning and guide lesion resection in all cases, no patient had new visual field deficits postoperatively.

**Conclusion:**

The proposed tractography strategy generates reliable and reproducible OR tractography images that can be reliably implemented in the routine, non-emergency pediatric neurosurgical setting.

## Introduction

The optic radiation (OR) is the primary visual white matter tract (WMT) in the human brain, connecting the lateral geniculate body (LGB) of the thalamus to the primary visual cortex in the occipital lobe (Ebeling and Reulen, 1988). Streamline tractography is a diffusion magnetic resonance imaging (MRI) post-processing technique that can generate connections closely following *in vivo* WMT fibers (Basser et al., 2000). It is increasingly being adopted in neurosurgical practice as an imaging adjunct, assisting with preoperative planning and intraoperative neuronavigation (Berman, 2009; Daga et al., 2012).

Injury to the OR results in visual field deficits (VFDs), the severity of which is determined by the retinotopic organization of the affected OR fiber bundle. The OR is typically described in three arbitrarily defined bundles based on classic cadaveric fiber dissection descriptions (Meyer, 1907; Ebeling and Reulen, 1988; Benjamin et al., 2014): The posterior bundle (PB) projects dorsally from the LGB, courses within the lateral ventricular wall WM – the sagittal stratum (SS) *intermediate* – before terminating in the superior calcarine cortex. The middle bundle (MB) leaves the LGB in a medial to lateral direction, crosses the temporal stem, then curves dorsally to terminate in the occipital pole. The anterior bundle/Meyer’s loop (ML) travels in a posteromedial to anterolateral direction from the LGB toward the temporal pole (TP), and at variable distances, loops back dorsally over the lateral ventricular roof. Leaving the temporal lobe, it courses within the inferior SS *intermediate*, before terminating in the inferior calcarine cortex. Injuries to the ML, PB and MB result in homonymous superior and inferior quadrant VFDs, and central (macular) VFDs, respectively.

VFDs due to surgical OR injuries are frequently reported in epilepsy and tumor neurosurgery (Hughes et al., 1999; Nilsson et al., 2004). In adult anterior temporal lobectomy series, the frequency of superior quadrant VFD due to ML injuries ranges from 50 to 100% (Hughes et al., 1999; Nilsson et al., 2004). Temporal lobe epilepsy surgeries guided by preoperative OR tractography have been shown to reduce the frequency of postoperative VFDs (Cui et al., 2015; Lilja et al., 2015).

Despite its current clinical utility, inconsistencies exist between the reconstructed OR tractography images and the anatomical ground-truth demonstrated by cadaveric dissection (Mandelstam, 2012; Benjamin et al., 2014). Resection surgeries informed by inadequate tractography techniques potentially result in permanent neurological deficits with associated functional impairments (Kinoshita et al., 2005; Duffau, 2014). In addition, streamline tractography is a complex post-processing procedure, involving many decisions by the operator based on combinations of protocol and anatomical knowledge.

The OR anatomy poses challenges for all tracking algorithms, with partial volume effects adjacent to ventricles (Mandelstam, 2012; Benjamin et al., 2014), high curvature in ML and multiple fiber populations in the temporal stem (Ebeling and Reulen, 1988; Sherbondy et al., 2008). Accordingly, it is difficult “…even when applying careful meticulous microtechniques, to separate a certain fiber system among this dense stratification and to guarantee an accurate and reliable anatomical differentiations, particularly over the sagittal stratum” (Yasargil et al., 2004).

The traditional diffusion tensor imaging (DTI) modeling approach assumes single fiber orientation within each MRI voxel, limiting the differentiation of fiber populations in crossing-fiber regions (Tournier et al., 2011). It has been demonstrated that combinations of a DTI model and a deterministic tractography algorithm typically underestimate the complexity of OR anatomy (Mandelstam, 2012; Benjamin et al., 2014). More advanced modeling of local diffusion signal can be applied by acquiring high angular resolution diffusion imaging (HARDI) data (Tournier et al., 2007, 2011). While such method can improve reconstruction results in regions containing multiple fiber populations, they remain under-utilized in OR tractography studies (Lim et al., 2015; Nowell et al., 2015; Bopp et al., 2019), particularly in pediatric neurosurgery (Chamberland et al., 2017). Probabilistic tracking algorithm applied to HARDI data is used in this study.

The placement of regions-of-interest (ROIs) is crucial to the anatomical accuracy of the targeted tractography, especially in clinical populations and in children. However, precise details of ROI placement are limited in existing OR tractography studies (Benjamin et al., 2014). It is therefore important to gain an understanding of variability of results between tractography operators and across different types of surgical cases. Some recent work has begun to address this issue. Results of a large number of tractography operators modeling the cortico-spinal tract in a single healthy subject using human connectome project data were compared in Rheault et al. (2019). There were other OR tractography studies addressing tracking reproducibility using healthy adult or typical developing pediatric MRI data (Nilsson et al., 2007; Chen et al., 2009; Dayan et al., 2015a; Chamberland et al., 2017). There remains a need to address the reliability of OR tractography methods in clinical pediatric populations (such as those with lesional epilepsy and brain tumor), given that most of the literature is both adult and healthy participant focused (Piper et al., 2014).

The contribution of this study is two-fold: Firstly, we provide a detailed description of a tractography protocol for delineation of the OR, suitable for advanced diffusion MRI acquisitions. We evaluated the proposed OR tractography strategy, that uses combined automated and manual ROIs, in the presence of a range of pathologies. The evaluation explores variability in tract delineation resulting from rater differences. Streamline tractography is performed using a higher order diffusion modeling approach based on HARDI data, combined with a probabilistic tracking algorithm; the former is used to resolve multiple fiber orientations within local voxels (Tournier et al., 2007, 2008), and the latter is used to model fiber orientation uncertainty in the form of a probability distribution rather than a single best-fit estimate used in a deterministic approach (Behrens et al., 2003). Secondly, we investigate the protocol repeatability with four tractography raters on clinically acquired, HARDI data from eight pediatric neurosurgical cases. These cases include a range of brain tumor and epilepsy pathologies and scans were acquired either preoperatively or intraoperatively. Reliability of the tracking method in both lesional and non-lesional hemispheres, from within a single (intra-rater) and between raters (inter-rater) were examined. This study should therefore provide important information concerning the use of advanced tractography in neurosurgical practice, with surgical patients and data that can be acquired clinically.

## Materials and Methods

### Study Participants

**FIGURE 1:**
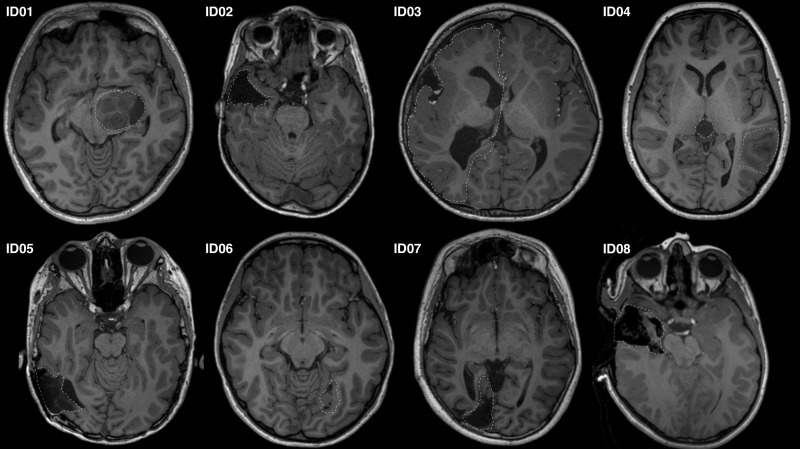
Representative axial T1-MRI images of all study participants. Images shown in radiologic convention. White dashed lines delineate the pathology/lesion or region of anatomical distortion involving the optic radiation.

Eight patients presenting to the children’s epilepsy surgery program at our institution were retrospectively selected via convenience sampling. Patients of different age and sex were carefully chosen with pathology and/or surgical changes at different locations along the OR (Figure 1). We studied seven preoperative and one intraoperative MRI data. Our institutional Human Research Ethics Committee approved the study as a clinical audit and determined informed consent was unnecessary. Patients’ clinical and imaging data were de-identified prior to analysis.

### MRI Scans

We acquired both HARDI diffusion and volumetric T1 structural images using either a diagnostic 3T MRI scanner with 32 channels head coil (Siemens Magnetom Trio Tim syngo MRI, Erlangen, Germany), or a 3T movable Siemens intraoperative MRI scanner with eight channels head coil (IMRIS, Manitoba, ON, Canada). The same acquisition sequences were used for both scanners equipped with maximum diffusion gradient strength of 40 mTm^–1^. The anatomical acquisition was T1-weighted high-resolution magnetization prepared gradient echo (MPRAGE) (320 × 320 acquisition matrix, FOV = 250 × 250 mm^2^, 0.78 mm^3^ isotropic voxels, TR/TE = 1900/2.69 ms, acquisition time = ∼6.5 min). HARDI diffusion data were acquired using a single-shot spin-echo echo-planar imaging readout using the following parameters: 60 non-collinear diffusion-weighted gradient directions, *b*-value = 3000 s/mm^2^, 7 *b*-value = 0 s/mm^2^ reference volumes, 54 contiguous slices, 2.3 mm^3^ isotropic voxels, TR/TE = 7600/110 ms, parallel acceleration factor = 2, acquisition time = ∼15 min.

### MRI Data Processing

Raw HARDI data were denoised (Veraart et al., 2016), and corrected sequentially for Gibbs-ringing artifacts (Kellner et al., 2016), motion and eddy current distortions (Smith et al., 2004), and b1 bias field inhomogeneity (Tustison et al., 2010). The diffusion gradient direction was updated based on the affine transformation used to perform motion and eddy current correction. A brain mask that combined T1-weighted image-based gray matter (GM) and white matter (WM) tissue segmentations was created using SPM8. The T1 images were linearly registered to the b0 diffusion images using the mutual information coregistration method in SPM8, and non-linearly using FSL FNIRT (Smith et al., 2004). The registration accuracy was visually inspected and confirmed between structural and diffusion images. The MRtrix3 software package^1^ (version 0.3.14) (Tournier et al., 2019) was used to estimate the fiber orientation distribution (FOD) at each preprocessed HARDI data voxel using constrained spherical deconvolution (CSD) with a maximum harmonic order (*l*_*max*_) = 8 (Tournier et al., 2007, 2008). A FOD-based directionally-encoded color (DEC) map was generated to guide ROI placements.

T1 structural images were processed using FreeSurfer, producing a parcelation of cortical and subcortical regions from which thalamus, peri-calcarine cortex ROIs were derived (see Supplementary Figure S1) (Desikan et al., 2006).

### Optic Radiation Tractography Methods

Probabilistic tractography was performed using the second order integration over FOD (iFOD2) (Tournier et al., 2010) algorithm in MRtrix3. The following default tractography parameters were used: 2500 streamlines per tract image; step size = 1.15 mm; maximum curvature = 45°; minimum and maximum track lengths = 11.5 and 230 mm; FOD amplitude threshold = 0.1. The reliability of these default tracking parameters was demonstrated previously (Tournier et al., 2012). They represent relatively conservative estimates available for neurosurgical applications, to ensure the tract of interest is delineated to its full extent.

A three-phase strategy was employed. The first phase identified the LGB, approximated by the optic tract (OpT) termination in the thalamus. The second phase delineated the superior component of the OR (PB and portions of MB fibers), terminating in the superior calcarine cortex. The third phase delineated the inferior component of the OR (combined ML and portions of MB fibers), terminating in the inferior calcarine cortex.

**FIGURE 2:**
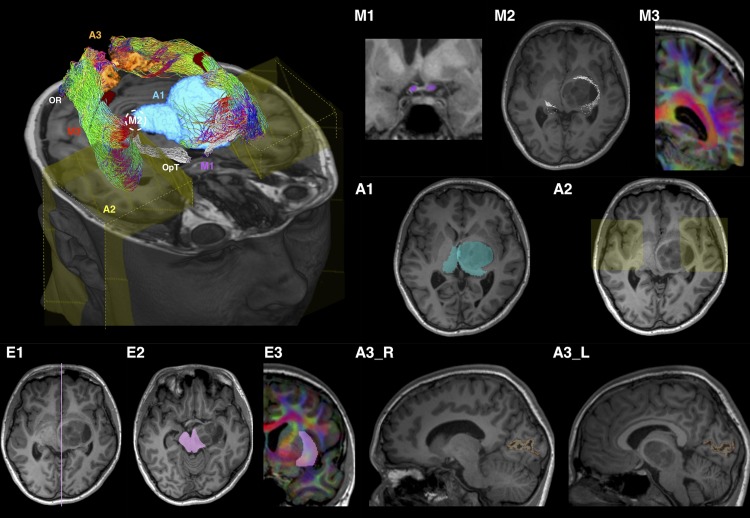
Optic radiation (OR) tractography regions-of-interest (ROI) summary, illustrated using patient ID01 MRI data (with a left thalamic tumor). The 3D volume rendered image is oriented by anatomic convention. The MRI images are oriented by radiology convention. The tractography method involves applying one manually defined seed ROI **(M2)**, two manually drawn inclusion ROIs (**M1** and **M3**), three automated inclusion ROIs **(A1–A3)**, and three manually drawn exclusion ROIs **(E1–E3)**. **M1**, optic tract (OpT); **M2**, lateral geniculate body (LBG); **M3**, internal and intermediate sagittal stratum; **A1**, right thalamus and left combined thalamus and tumor mask; **A2**, anterior temporal regions; **A3_R/L**, right/left peri-calcarine cortex; **E1**, sagittal midline; **E2**, midbrain at the level of inferior colliculi; **E3**, external and extreme capsules. The LGBs are marked by white dash circles in both the volume rendered and MR **(M2)** images. ROI **M3** and **E3** are delineated using the diffusion directionally-encoded color (DEC) images displayed in T1 resolution. In the volume rendered image, the OR tractography is color-coded by tract directions, same as the DEC images: left-right (red), superior-inferior (blue), and anterior-posterior (green). The OpT tractography is shown in white color.

Tracking employed *seed*, *inclusion* and *exclusion* ROIs (see Supplementary Document for detail definitions). The ROI placements are summarized in Figure 2 and described below.

#### Phase 1 – LGB Identification

Tracking was performed from the *OpT seed* ROI (Figure 2-M1) toward the *thalamus inclusion* ROI (Figure 2-A1). The mid-point between the terminations of OpT streamlines and the posterior thalamic border at the level of the diencephalon-mesencephalon junction was used as the approximate location of the LGB.

#### Phase 2 – Superior Component of OR

A 5 mm radius sphere, centered on the LGB location determined in Phase 1, was used as a *seed* ROI (Figure 2-M2). The peri-calcarine cortex (Figures 2-A3_R, A3_L) and an SS inclusion ROI delineated manually (Figure 2-M3) were the *inclusion* ROIs (see Supplementary Document for detail descriptions). Three *exclusion* ROIs were manually defined: at the midline (Figure 2-E1); at the midbrain (Figure 2-E2), and at the external and extreme capsules (Figure 2-E3).

#### Phase 3 – Inferior Component of OR

An anterior temporal (AT) inclusion region (Figure 2-A2) was included in addition to those used in Phase 2.

#### Modifications for Lesional Hemisphere

ROI placement was modified when required by pathology. Most changes were related to partial failures of automated tools due to pathology or previous surgery. The modifications were conservative and are described in detail in Supplementary Document.

#### Post-tractography Streamlines Editing

Classic cadaveric fiber dissection images from the Ludwig and Klinger’s atlas (Ludwig and Klingler, 1956) were used as anatomical ground truth to guide removal of spurious streamlines. While this atlas is in itself a subjective method of studying OR anatomy and suffers from limitations relating to specimen preparation and dissection resolution at a macroscopic scale, fiber dissection studies remain a widely accepted method referencing WM anatomy.

#### Tract Masks

Post-edited streamlines from both the superior and inferior OR components were merged together and converted to an overall OR tract mask via track density imaging. All quantitative imaging metrics used for the test-retest assessment were derived from these tract masks.

### Intra- and Inter-Rater Reliability/Agreement Analysis

#### Tractography Rater Profiles

Four tractography raters with a variety of clinical and imaging backgrounds participated in this study: (a) **Rater A:** 6 years neurosurgical practice and tractography experience; (b) **Rater B:** 4 years general pediatric radiology practice with no prior tractography experience; (c) **Rater C:** imaging scientist with no clinical background, and minimal tractography experience; (d) **Rater D:** 8 years clinical neuropsychology practice, experienced in functional MRI processing and 4 years of tractography experience. Apart from rater A, who was responsible for the study design, tractography raters were blind to patient clinical information. Prior to performing the tractography, rater B to D studied an in-house document, including accounts of the OR anatomy with images from the Ludwig and Klinger’s atlas and step-by-step instructions on generating the manual ROIs, and performing post-tracking editing. All raters followed the same strategy for ROI placements, as described above.

#### Intra- and Inter-Rater Tests

All raters performed tractography independently for all cases at least once. Rater A and B, with the most and the least tractography experience, performed all tractography twice with approximately an interval of 4 months between the attempts, providing a basis for intra-rater comparisons.

#### Tractography Metrics

Diffusion metrics including mean apparent fiber density (AFD), fractional anisotropy (FA), and mean diffusivity (MD) were computed for the OR masks. Tract volumes (TV), the 3D distances between the temporal pole and anterior edge of ML (i.e., TP-ML distances), and between the anterior edges of both temporal ventricular horn and ML (i.e., TH-ML distances) were derived from the OR masks. We additionally computed the 2D TH-ML distance based on the sagittal plane (i.e., *y-axis* coordinates) given it is frequently reported by other OR tractography studies, thus allowing for more direct study comparisons (see Supplementary Document for detail descriptions).

### Statistical Analysis

Statistical analysis was performed using the *R* software environment for statistical computing and graphics (R Core Team; 2013). All analyses were summarized by hemispheric sides and then by lesional versus non-lesional OR.

The outcomes of OR tractography between raters were assessed using the intraclass correlation coefficient (ICC) to represent the degree of absolute agreement between *k* randomly selected judges. The ICC coefficients were computed using the tractography metrics described above. For the LGB coordinates, the ICC coefficients were computed using the Euclidean distance of the LGB from center of the brain. ICC coefficients were interpreted as *poor* (<0.40), *fair* (0.40 ≥ ICC < 0.60), *good* (0.60 ≥ ICC < 0.75), and *excellent* (ICC ≥ 0.75). Only the first tractography attempts of all four raters were included for the analysis (i.e., the repeat tractography attempts by rater A and B were excluded to avoid bias).

The degree of spatial agreement in binary tract masks was assessed using the median dice similarity coefficient (DSC). The DSC is defined as: DSC = $2\,\frac{\left|X\right|\cap \left|Y\right|}{\left|X\right|+\left|Y\right|}$, where X and Y are the number of voxels in each tract mask. The DSC ranges from 0, indicating no overlap in the two masks, and 1, indicating perfect overlap between the masks. DSC values were interpreted as *poor* (<0.40), *fair* (0.40 ≥ DSC < 0.60), *good* (0.60 ≥ DSC < 0.75), and *excellent* (DSC ≥ 0.75). For each tract, all pair-wise DSCs between raters were calculated. The median DSC across raters was then computed.

### Visualization

Per patient consensus OR tract masks, serving as a study-specific OR anatomical “ground-truth,” were derived from all raters’ OR tractography images (Warfield et al., 2004). Distances between each tract mask and a consensus mask were computed and displayed on the individual meshes to provide a representation of spatial distribution of disparities.

## Results

**FIGURE 3:**
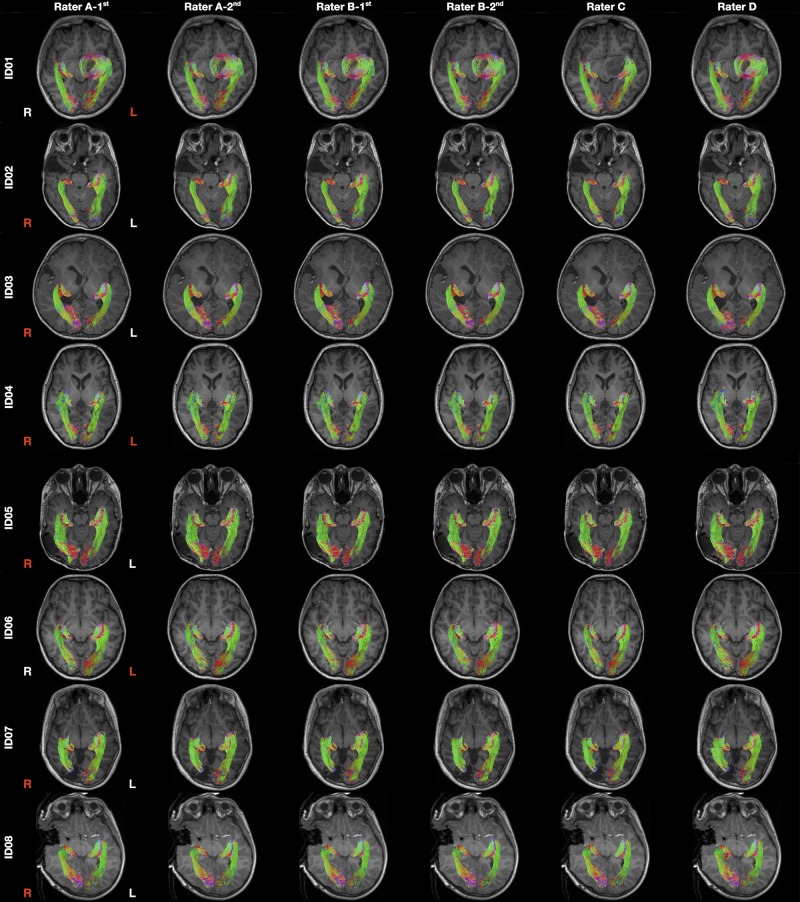
Final optic radiation tractography images generated from all raters. The tractography images are color coded by tract directions: left-right (red), superior-inferior (blue), and anterior-posterior (green). The MRI images are displayed in radiology convention. L, left; R, right. Rater A (B)- first/second = first/second tractography attempt by rater A (B). Hemisphere side colored by lesion/pathology: red, lesional; white, non-lesional.

Patient demographics and clinical information are summarized in Table 1. Median patient age was 12.2 years old (ranged 2.7–16.5 years old). There were six males. The OR tractography images derived from all tracking attempts prior to *the streamline editing* step are provided in Supplementary Figure S2. The final tractography images are shown in Figure 3. Data processing time is summarized in Supplementary Table S1.

**TABLE 1:** Demographic and imaging features of the study participants.

**ID**	**Lesion**				**Goldmann VF perimetry (Y/N)**	**Goldmann VF perimetry findings**
			
	**Epilepsy/Tumor**	**Side**	**Description and Location (Optic radiation component involved)**	**Pathology**		
1	Tumor	L	Expanding solitary thalamic lesion (LGB)	Pilocytic astrocytoma	N	Intact (on confrontation)
2	Tumor	R	Previous R anterior temporal lesionectomy (ML)	Recurrent anaplastic ependymoma	Y	Left HSQ-VFD^∗^
3	Epilepsy	R	Small, deformed thalamus. Extensive frontal, temporal and parietal cortical thickening and SHE; *Exca vaco* ventricular dilatation (entire OR)	Hemispheric FCD sparing occipital lobe	N	Intact (on confrontation)
4	Epilepsy	L	Expanding angular gyrus lesion, displacing and infiltrating into the SS (Principally PB in the SS)	Protoplasmic astrocytoma	Y	Intact
				
		Mid	Midline pineal lesion (LGB)	Pineal region colloid cyst		
5	Epilepsy	R	Gliosis of the fusiform and inferior temporal gyrus (Principally ML in the SS)	FCD and gliosis secondary to neonatal cerebral hemorrhage	Y	Intact
6	Epilepsy	L	Expanding lingual gyrus lesion, displacing the inferior calcarine cortex (ML and MB)	Pleomorphic Xanthoastrocytoma	Y	Intact
7	Epilepsy	R	Previous occipital lobectomy with minimal residual occipital lobe showing atrophic changes (entire OR; missing PCCx)	FCD and gliosis due to neonatal ischemic vascular injury	Y	Left HH-VFD^∗^
8	Epilepsy	R	Anterior temporal gliosis; previous tumor resection (ML)	Gliosis	Y	Left HSQ-VFD^∗^

*FCD, focal cortical dysplasia; HH, homonymous hemianopsia; HSQ, homonymous superior quadranopsia; L, left; MB, middle bundle; ML, Meyer’s loop (anterior bundle); Mid, midline; N, no; PCCx, peri-calcarine cortex; PB, posterior bundle; VFD, visual field deficits; R, right; SHE, subependymal heterotopia; SS, sagittal stratum; Y, yes. ^∗^Pre-existing visual field deficits from previous epilepsy surgeries.*

### ICC and DSC Scores

**FIGURE 4:**
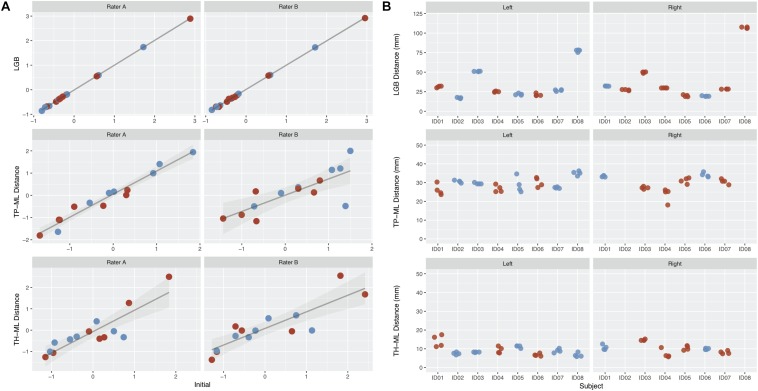
The intra-rater and inter-rater reliability and agreement performance from all raters derived optic radiation (OR) morphology metrics. **(A)** Intra-rater test-retest results for rater A and B. Metrics derived from the initial tracking is shown on the *x*-axis, with repeated tracking shown on the *y*-axis. Values represent *z-*scores. **(B)** Inter-rater test-retest results from all four raters. The patients are represented on the *x*-axis, with each point representing a different rater. Red points, metrics derived from the lesional OR; blue points, metrics derived from the non-lesional OR. Note there are no TP-ML and TH-ML metrics available for right lesional OR derived from patient ID02 and ID08 due to the absence of right temporal poles in both cases. LGB, lateral geniculate body; TP-ML, 3D distance between the temporal pole and Meyer’s loop; TH-ML, 3D distance between the temporal horn of lateral ventricle and Meyer’s loop.

Overall, there were eight patients and MRI datasets, four tractography raters, and 128 tractography metrics were used to calculate the ICC and DSC scores. The intra- and inter-rater ICCs and median DSC values are summarized in Tables 2, 3 and Figure 4 (see also Supplementary Figures S3, S4).

**TABLE 2:** Intra-rater ICC and DSC scores in both hemispheres.

	**Left Hemisphere**	**Right Hemisphere**
**Rater A**		
ICC volume	0.99 [0.95, 0.99]	0.98 [0.82, 0.99]
ICC AFD	0.99 [0.99, 0.99]	0.82 [0.38, 0.96]
ICC FA	0.99 [0.99, 0.99]	0.99 [0.99, 0.99]
ICC MD	0.99 [0.99, 0.99]	0.99 [0.99, 0.99]
DSC	0.89	0.89
ICC LGB	0.99 [0.99, 0.99]	0.99 [0.99, 0.99]
ICC TP-ML	0.98 [0.90, 0.99]	0.96 [0.78, 0.99]
ICC TH-ML	0.76 [0.25, 0.95]	0.93 [0.65, 0.99]
**Rater B**		
ICC volume	0.98 [0.93, 0.99]	0.94 [0.73, 0.99]
ICC AFD	0.99 [0.99, 0.99]	0.99 [0.99, 0.99]
ICC FA	0.97 [0.86, 0.99]	0.99 [0.99, 0.99]
ICC MD	0.99 [0.99, 0.99]	0.99 [0.99, 0.99]
DSC	0.89	0.89
ICC LGB	0.99 [0.99, 0.99]	0.99 [0.99, 0.99]
ICC TP-ML	0.88 [0.56, 0.98]	0.91 [0.56, 0.99]
ICC TH-ML	0.90 [0.61, 0.98]	0.81 [0.22, 0.97]

*DSC is median DSC across all pairwise comparisons between raters. ICC coefficients were computed using a one-way consistency model for intra-rater comparisons, and two-way agreement for inter-rater comparisons. 95% confidence intervals are given in square brackets. Abbreviations: AFD, apparent fiber density; DSC, dice similarity coefficient; MD, mean diffusivity; FA, fractional anisotropy; ICC, intraclass coefficient; LGB, lateral geniculate body; TH-ML, distance between the tip of temporal ventricular horn and the anterior edge of Meyer’s loop; TP-ML, distance between the temporal pole and the anterior edge of Meyer’s loop.*

**TABLE 3:** Inter-rater ICC and DSC scores in both hemispheres.

	**Left**	**Right**
**All**		
ICC volume	0.76 [0.48, 0.94]	0.89 [0.57, 0.98]
ICC AFD	0.99 [0.99, 0.99]	0.99 [0.99, 0.99]
ICC FA	0.99 [0.97, 0.99]	0.99 [0.98, 0.99]
ICC MD	0.99 [0.98, 0.99]	0.99 [0.99, 0.99]
DSC	0.88	0.86
ICC LGB	0.99 [0.99, 0.99]	0.99 [0.99, 0.99]
ICC TP-ML	0.72 [0.41, 0.92]	0.81 [0.53, 0.97]
ICC TH-ML	0.74 [0.45, 0.93]	0.80 [0.51, 0.97]
**Lesion hemisphere**		
ICC volume	0.76 [0.28, 0.99]	0.89 [0.51, 0.98]
ICC AFD	0.99 [0.95, 0.99]	0.99 [0.99, 0.99]
ICC FA	0.90 [0.52, 0.99]	0.99 [0.98, 0.99]
ICC MD	0.94 [0.71, 0.99]	0.99 [0.99, 0.99]
DSC	0.84	0.87
ICC LGB	0.95 [0.75, 0.99]	0.99 [0.99, 0.99]
ICC TP-ML	0.41 [0.21, 0.97]	0.70 [0.25, 0.97]
ICC TH-ML	0.74 [0.13, 0.99]	0.85 [0.53, 0.99]
**Non-lesion hemisphere**		
ICC volume	0.92 [0.71, 0.99]	0.97 [0.75, 0.99]
ICC AFD	0.99 [0.98, 0.99]	0.98 [0.83, 0.99]
ICC FA	0.99 [0.96, 0.99]	0.94[0.59, 0.99]
ICC MD	0.99 [0.99, 0.99]	0.98 [0.87, 0.99]
DSC	0.87	0.88
ICC LGB	0.99 [0.99, 0.99]	0.99 [0.99, 0.99]
ICC TP-ML	0.89 [0.64, 0.99]	0.98 [0.90, 0.99]
ICC TH-ML	0.82 [0.50, 0.98]	0.99 [0.99, 0.99]

*DSC is median DSC across all pairwise comparisons between raters. ICC coefficients were computed using a one-way consistency model for intra-rater comparisons, and two-way agreement for inter-rater comparisons. 95% confidence intervals are given in square brackets. AFD, apparent fiber density; DSC, dice similarity coefficient; MD, mean diffusivity; FA, fractional anisotropy; ICC, intraclass coefficient; LGB, lateral geniculate body; TH-ML, distance between the tip of temporal ventricular horn and the anterior edge of Meyer’s loop; TP-ML, distance between the temporal pole and the anterior edge of Meyer’s loop.*

### TP-ML and TH-ML Distances

**FIGURE 5:**
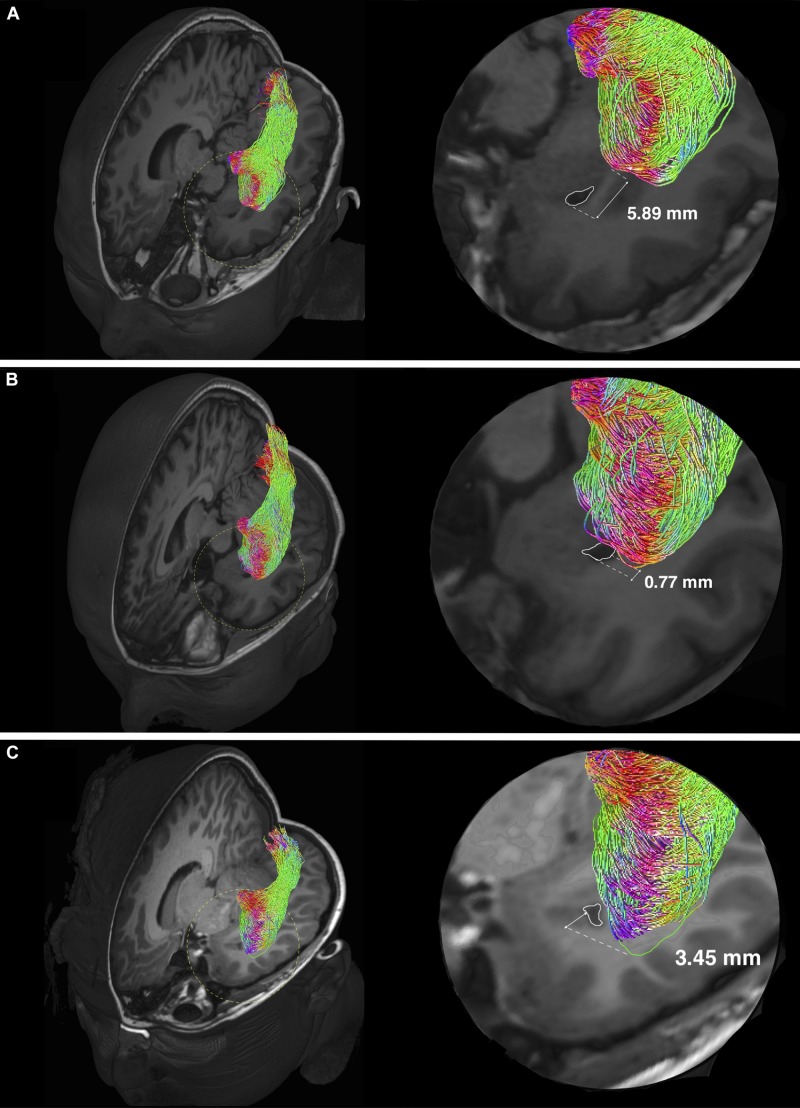
Individual variability of the Meyer’s loop position in temporal lobe. Optic radiation tractography examples showing variations of the anterior bundle/Meyer’s loop (ML) position, relating to the temporal ventricular horn (TH) (delineated by white colored lines). Magnified views (delineated by yellow colored dashed circles) are shown on the left. The anterior edge of AB/ML can be behind **(A)**, approximately at the level **(B)** and in front **(C)** of the TH. The TH-ML distance is expressed in millimeters. In panel **(C)**, the green colored line delineates the anterior edge of AB/ML, which is slightly out-of-plane.

The mean TP-ML and TH-ML distances calculated from all ORs reconstructed with intact temporal lobe (*n* = 14) are summarized in Table 4. The mean ML position was approximately 30 mm from the TP, and 9 mm anterolateral to the tip of TH. In the 2D sagittal plane, the ML position ranged from approximately 5 mm behind of to 10 mm in front of the tip of TH. Figure 5 demonstrates individual variations of the ML position.

**TABLE 4:** Summary of the Meyer’s loop positions from temporal anatomical landmarks.

	**3D TP-ML distance (mm)**	**3D TH-ML distance (mm)**	**2D TH-ML distance (mm)**
All OR (*N* = 14)^∗^	29.39 ± 3.52 95% CI 0.75 [18.08–36.25]	9.39 ± 2.62 95% CI 0.56 [5.67–17.5]	1.35 ± 2.90 95% CI 0.62 [(−5.89) –10.26]
Lesional OR (*N* = 7)^∗^	27.77 ± 3.04 95% CI 0.92 [18.08–32.70]	9.91 ± 3.31 95% CI 1.00 [5.67–17.5]	2.17 ± 2.99 95% CI 0.91 [(−2.89) –10.26]
Non-lesional OR (*N* = 7)	31.02 ± 3.27 95% CI 0.99 [23.40–36.25]	8.88 ± 1.59 95% CI 0.48 [5.85–12.56]	0.52 ± 2.61 95% CI 0.79 [(−5.89) –5.35]

*Measurements are reported in mean ± standard deviation, followed by 95% confidence interval (CI), and range in square brackets. Minus sign represents the Meyer’s loop (ML) position behind of the temporal ventricular horn. mm, millimeter; OR, optic radiation; TH-ML, distance between the tip of temporal ventricular horn and the anterior edge of Meyer’s loop; TP-ML, distance between the temporal pole and the anterior edge of Meyer’s loop. **^∗^**Excluding the two right lesional ORs (patient ID 02 and ID08) without temporal poles.*

### Consensus OR Tract Masks Visualization

**FIGURE 6:**
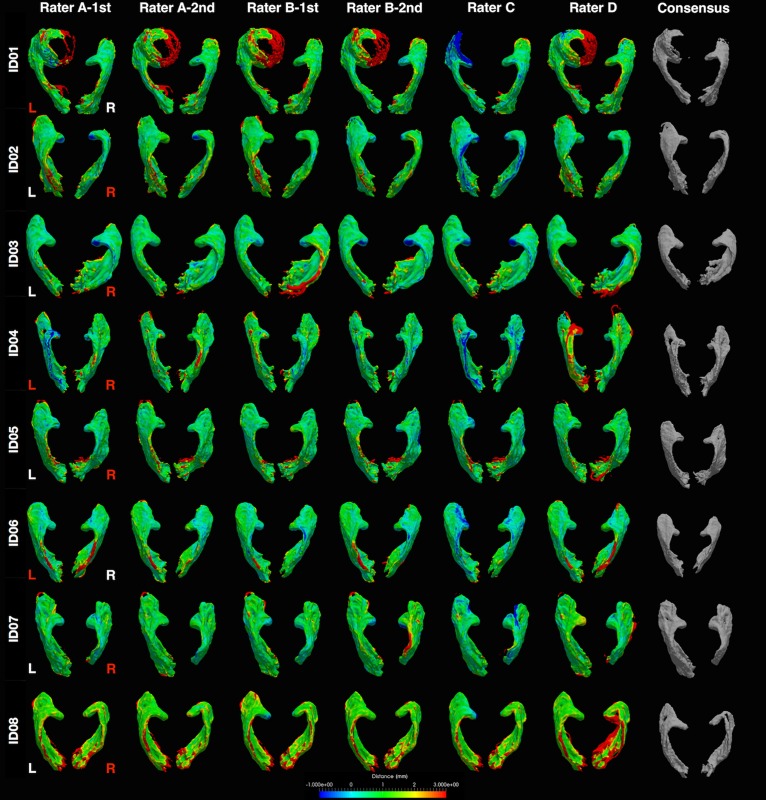
Distance heat maps of individual optic radiation tract mask from the consensus masks. Images displayed in anatomical convention. L, left; R, right. Rater A (B)- first/second = first/second tractography attempt by rater A (B). Hemisphere side colored by lesion/pathology: red, lesional; white, non-lesional. mm, millimeter.

The distance maps computed between all tracking attempts from the consensus masks are displayed in Figure 6. Overall, the distance variations between different tracking attempts were mostly within ± 1 mm of the consensus masks, with only few exceptions (e.g., left OR in patient ID01).

### Clinical Utility of the OR Tractography

The OR tractography results correlated well with preoperative Goldmann VF perimetry findings in six patients undergoing the test (Table 1). The preoperative OR images were used to assist surgical planning and guide resection in all cases. While such an approach was limited in itself unable to correct for real-time navigation inaccuracy due to intraoperative brain shift, no patient had new postoperative VFDs. In the youngest patient (ID03), the tractography images were used to complement the inter-ictal Positron Emission Tomography findings, showing tract terminations in metabolically active (thus likely functionally normal) visual cortex. Surgery was performed to resect metabolically inactive frontal and parietal cortex, sparing the functional occipital lobe and the OR, instead of performing hemispheric disconnection surgery, which would result in permanent homonymous VFDs.

## Discussion

Our method employs a combination of manual and automatically generated ROIs to guide OR tractography followed by manual cleanup of spurious streamlines. We demonstrate that following training, and despite some distinctions between manually defined ROIs within and between raters with different tractography experience, the outcomes of OR tractography were highly consistent. Our test-retest results are comparable with reporting by other FOD-based OR tractography studies using either healthy participants (Dayan et al., 2015a) or adult epilepsy patients (Nowell et al., 2015). Reliability was generally lower in hemispheres with pathology than without, as expected. The measure with lowest DSC score (TP-ML distance, left lesional hemisphere) was driven by a combination of low patient numbers and an inconsistent measure from the least experienced rater.

The tractography results closely resemble classical descriptions of OR anatomy derived from dissection studies, with a continuous “sheet-like” structure (Figure 3). Our reported OR tract volumes (mean ± standard deviation = 22.66 ± 4.24 cm^3^) are slightly larger than those derived from myelin-stained histology using adult cadaveric brain specimens (mean ± standard deviation = 18.4 ± 2.10 cm^3^) (Bürgel et al., 1999). Our ML position results closely match with classic dissection studies: mean 3D TP-ML distance = 29.4 mm (range 18.08–36.25 mm) in our study versus 27.35 mm (range 15–37 mm) in dissection studies; mean 2D TH-ML distance = 1.35 mm [range (−5.89)–10.26 mm] in our study versus 3 mm in dissection studies [range (−5)–10 mm] (Ebeling and Reulen, 1988; Peuskens et al., 2004; Rubino et al., 2005; Choi et al., 2006; Chowdhury and Khan, 2010). Our reported 3D TH-ML distance range (5.85–12.56 mm) corresponds closely with an observation made by classic neuroanatomist, Moritz Probst, while studying the Marchi-stained degenerated OR fibers, describing the anterior portion of ML is located 5–10 mm lateral to the tip of TH and amygdala (Yasargil et al., 2004). A future *ex vivo* diffusion MRI study utilizing post-mortem brain may better inform the anatomical validity of our OR tractography method, and is of great importance in the field of diffusion MRI research. However, applicability of such findings to the *in vivo* clinical (particularly surgical) scenario would be limited due to differences in the scanning environment, sequence selections, and brain tissue characteristics.

Our results are generally comparable with those using probabilistic FOD-based tractography (Lim et al., 2015; Nowell et al., 2015) and compare favorably to studies utilizing deterministic DTI-based tractography (Nilsson et al., 2007; Chen et al., 2009), which typically under-represent the anterior extent of ML fibers. Probabilistic DTI-based studies (Sherbondy et al., 2008; Dayan et al., 2015b), or those utilizing more advanced DTI modeling approaches (Winston et al., 2011; Lim et al., 2015), and deterministic FOD-based studies (Chamberland et al., 2017) variably recover the anterior ML fibers, and also generally perform better than the traditional deterministic DTI-based studies.

There are only two previous pediatric OR tractography studies, both using typical developing cohorts (Dayan et al., 2015b; Chamberland et al., 2017). Our reported ML positions differ from these studies, likely reflecting the differences in diffusion data acquisition type, tracking method, and inter-subject anatomical variability. In a pediatric subset (4 patients) of one tumor/epilepsy surgery series, the TP-ML distance reported was similar to ours (Chen et al., 2009).

We postulate the following explanations for our OR volumes and the ML positions being concordant with adult dissection studies. The OR commences and reaches complete myelination maturation (thus, reaching adult morphology) as early as 4 months postnatal life (Yakovlev and Lecours, 1967). While the majority of intracranial volume and ventricular volume growth occurs during brain maturation, especially in the first 15 years of life (Sgouros et al., 1999; Xenos et al., 2002), the relative rate of growth between the two remains stable throughout childhood (Xenos et al., 2002). Neuronal remodeling through synaptic pruning during puberty and young adulthood and disease/aging-related axonal degeneration might account for the volume discrepancy observed between our study and adult cadaveric studies (Luo and O’Leary, 2005).

The processing time required for our method currently precludes application in acute neurosurgical settings, though OR tractography is rarely utilized emergently. We have not addressed all possible retest scenarios, such as repeated scans of the same patient. Our study aimed to deliver a reliable tractography framework using the DWI and structural data only, thus we did not incorporate functional MRI activated cortical regions into our ROI placement strategies, which may improve the biological relevance of our tracking results. Conducting and obtaining reliable fMRI results may not be possible in poorly compliant and young children. Since the study completion, our institution’s clinical tractography processing had upgraded to incorporate a more advanced FOD modeling method based on multi-shell diffusion data, acquired using a time efficient multiband acquisition scheme. This imaging initiative may lead to further improvements in our tractography results. Finally, to address the proposed study objective, the intention of this study was not to recruit a large sample cohort of identical clinical cases; rather, limited numbers of cases were carefully selected to cover a variety of clinical scenarios.

## Conclusion

We presented and validated a practical tractography framework that can reliably delineate both morphologically normal and pathology-affected OR fiber tracts in children undergoing epilepsy and brain tumor surgeries. Reproducible and anatomically accurate results can be obtained with minimal training required, regardless of the rater’s clinical and research experiences. The excellent postoperative outcomes of patients involved in this study suggest that our tractography technique can be safely implemented in the routine, non-emergency pediatric neurosurgical setting.

## Data Availability Statement

All datasets generated for this study are included in the article/Supplementary Material.

## Ethics Statement

The studies involving human participants were reviewed and approved by the Human Research Ethics Committee of The Royal Children’s Hospital. Written informed consent from the participants’ legal guardian/next of kin was not required to participate in this study in accordance with the national legislation and the institutional requirements.

## Author Contributions

JY and RB: conception and design. JY, RB, SB, CM, and MW: data acquisition. JY, RB, CM, and C-HY: drafting the article. All authors: analysis and interpretation of the data, and critically revising and final approval of the manuscript. AH, VA, WM, and MS: study supervision.

## Conflict of Interest

The authors declare that the research was conducted in the absence of any commercial or financial relationships that could be construed as a potential conflict of interest.

## Footnotes

1 http://www.mrtrix.org
