# Supplementary material for: Optic Radiation Tractography in Pediatric Brain Surgery Applications: A Reliability and Agreement Assessment of the Tractography Method
Source: Front Neurosci. 2019 Nov 20;13:1254. doi: 10.3389/fnins.2019.01254 (PMC6879599; doi:10.3389/fnins.2019.01254)
Supplement: Supplementary file 1 [file Data_Sheet_1.PDF]

Supplemental Document

Tractography regions-of-interest (ROI) definitions

Tractography using MRtrix3 supports the use of *seed*, *inclusion* and *exclusion* ROIs. Tracking of streamlines commences from random locations within the *seed* ROI. Streamlines can be unidirectional or bidirectional, with bidirectional streamlines exiting the *seed* ROI in two directions. Streamlines intersecting an *exclusion* ROI are discarded. Streamlines can be terminated (i.e. not tracked further) if they reach a voxel with sub-threshold FOD values, exit a mask region, or if all inclusion regions have been traversed and a "*stop*" option was provided.

Detail descriptions of the following inclusion ROI make reference to Figure 2 in the main text.

SS inclusion ROI delineation

The *SS inclusion* ROI (Figure 2-M3) was manually delineated at the level of the ventricular atrium on one coronal T1 slice. The DEC map was used to assist identifying different SS components- the *external* (blue-purple color), *intermediate* (green color) and i*nternal* (blue-purple color) components. The OR travels within the SS *intermediate*. The ROI included both the *intermediate* and *internal* SS (due to the *internal* SS being a very thin structure, it is difficult to distinguish from the *intermediate* SS), but not the external SS. The ROI should also include the WM of the ventricular floor, housing the ML at this level.

AT inclusion ROI delineation

The *AT inclusion* ROI (Figure 2-A2) consisted of a "rectangular box" defined semi-automatically in 3D space. The center coordinates of LGB defined the dorsal-medial-superior

corner of the box. The *y-axis* of the box was parallel to the AC-PC line, *x-axis* parallel to the line between anterior temporal fossa tips and the *z-axis* was perpendicular to both. The AC-PC line and anterior fossa tips were defined manually. The y extent of the box was between LGB and anterior fossa, the z extent was from the LGB to the inferior brain edge and the x-extent was from LGB to outer brain edge. The outer and inferior edges of the brain were defined automatically by limits of the T1-based binarized brain mask.

Modifications for lesional hemisphere

Manual *SS inclusion* ROIs were placed on adjacent imaging slices with recognizable structural anatomy (patient ID04, and ID05). In patient ID07 with absent right occipital lobe, tracking was performed without using the *peri-calcarine cortex inclusion* ROI. For the lesional *LGB seed* ROI (patient ID01), pathology mask was combined with the *thalamus* ROI (Figure S1- ID01; left thalamus ROI), serving as termination *inclusion* ROI for optic tract tractography. Presence of the midline colloid cyst in patient ID04 did not affect the thalamus parcellation by FreeSurfer (Figure S1- ID04; thalamus ROI). In cases with erroneous FreeSurfer cortical parcellation scheme (patient ID03, and ID08), parcellations containing the peri-calcarine cortex were merged together and used as the occipital *inclusion* ROI for the final OR tractography (Figure S1- ID03 and ID08; right peri-calcarine ROI). No modifications were required for placements of the automated *AT inclusion* ROIs in anterior temporal lesionectomy cases (patient ID02, and ID08).

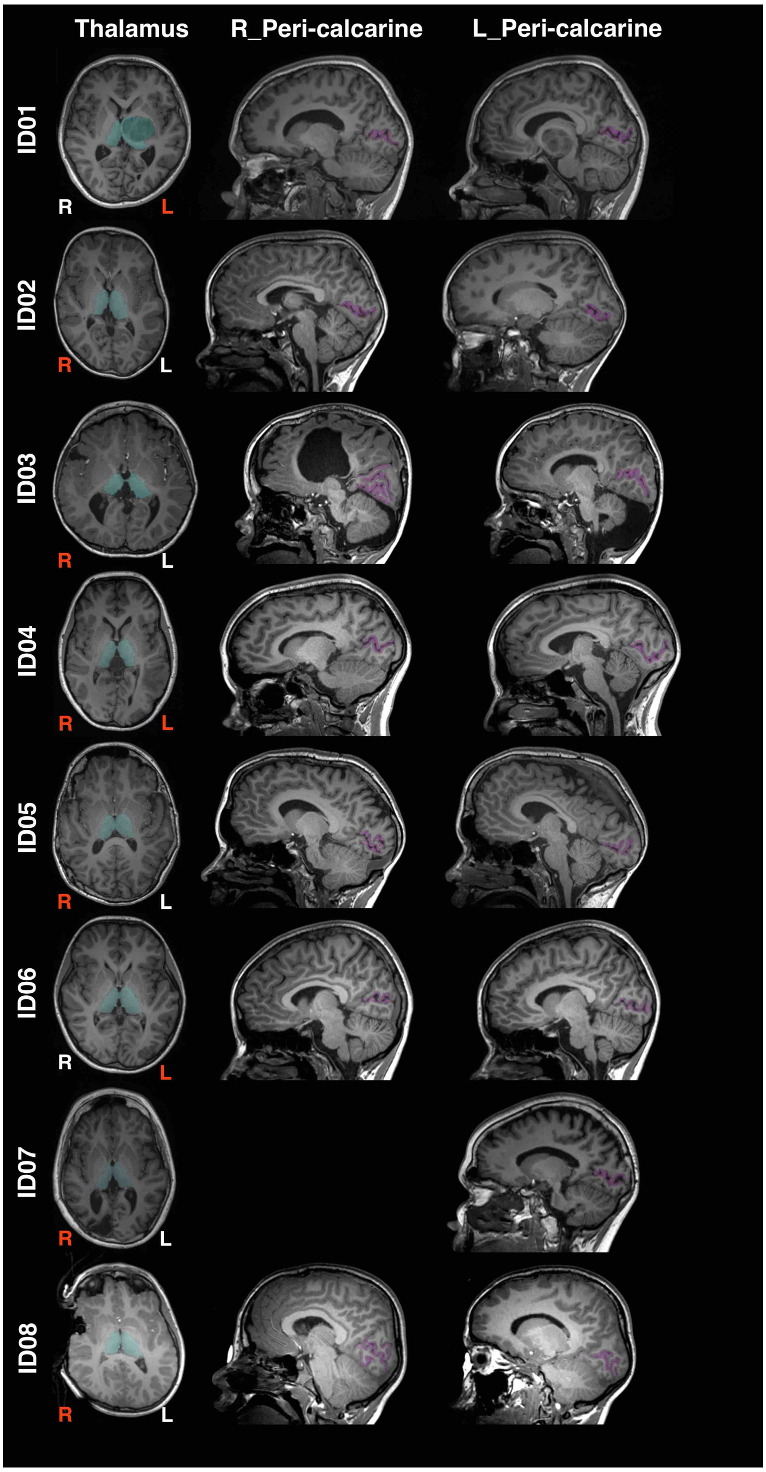

Figure S1. The thalamic and peri-calcarine inclusion regions of interest used in this study. These regions are modified based on automated FreeSurfer parcellation scheme using the Desikan-Killiany Atlas. The thalamus regions of interest (ROI) are in blue, and the peri-calcarine ROI are in pink. Hemisphere side colored by lesion/pathology: red = lesional; white = non-lesional. Abbreviations: R = right; L = left.

Calculation of the TP-ML and TH-ML distances

Approximate positions of left and right temporal pole, anterior (AC) and posterior commissures (PC) were manually determined and used in the following procedures. In addition, an automatically generated GM and cerebrospinal fluid (CSF) masks were used. The AC-PC vector was used to define the anterior-posterior vector. The position of landmarks used for anatomical measures were identified as follows:

- *Temporal pole (TP)*: the manually selected position was refined by searching a 15 voxel radius for the most anterior GM voxel.
- *Temporal horn (TH):* position of the closest CSF ventricle voxel to the temporal pole. A series of morphological operations were used to separate ventricle and peripheral CSF.
- *Meyers loop (ML)*: the voxel in the tract mask closest to the refined temporal pole position.

These positions were used directly to compute 3D Euclidean distances between landmarks. 2D versions were computed by projecting the vector onto the sagittal plane.

Supplemental Data

Table S1. Image processing time summary

	Processing time	Comments
**Automated processing steps**		
Diffusion data preprocessing	~ 60 minutes per case	Includes denoize, distortion corrections, image registration, FOD estimation and CSD modeling, and generating diffusion metric maps and FOD-DEC map
FreeSurfer parcellations	~ 420 minutes per case	
Probabilistic CSD tracking	~30 minutes per case	Includes processing for both OR from each brain
**Manual processing steps**		
Method training	Mean 10.7 minutes per case [Range 8 – 13 minutes]	Study anatomy and method document
Manual ROI placements	Mean 16.52 minutes per case [Range 10 – 29 minutes]	Include ROI steps M1-M3, and E1-E3 outlined in Figure 2
Streamline editing	Mean 35.7 minutes per case [Range 15 – 75 minutes]	

Abbreviations: FOD = fiber orientation distribution; CSD = constrained spherical deconvolution; DEC = directionally encoded color map; OR = optic radiation; ROI = regions of interest.

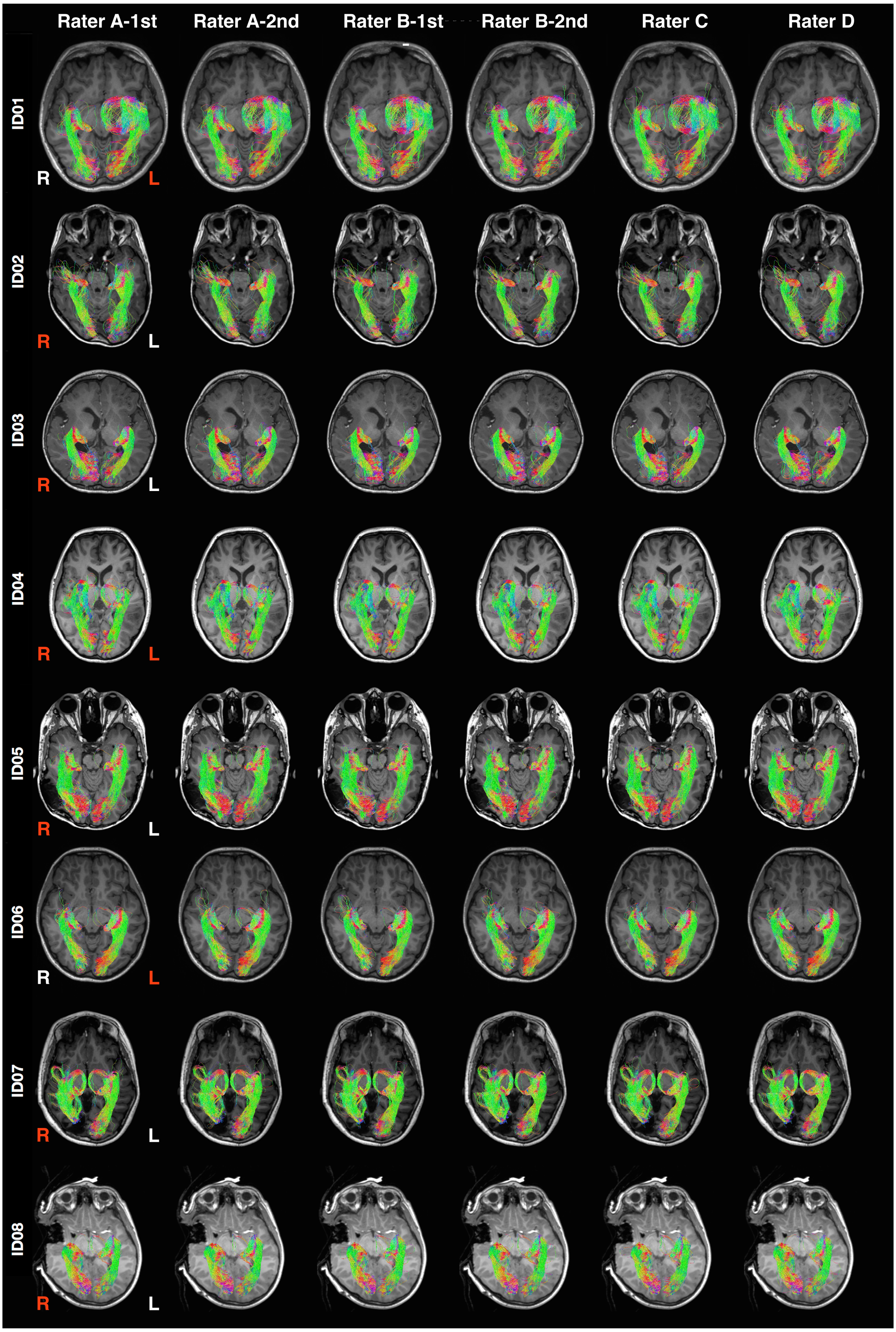

Figure S2. Intermediate optic radiation tractography images prior to streamline editing. The tractography images are color coded by tract directions: left-right (red), superior-inferior (blue), and anterior-posterior (green). The MRI images are displayed in radiology convention. L = left; R = right. Rater A(B)-1st/2nd = first / second

tractography attempt by rater A(B). Hemisphere side colored by lesion/pathology: red = lesional; white = non-lesional.

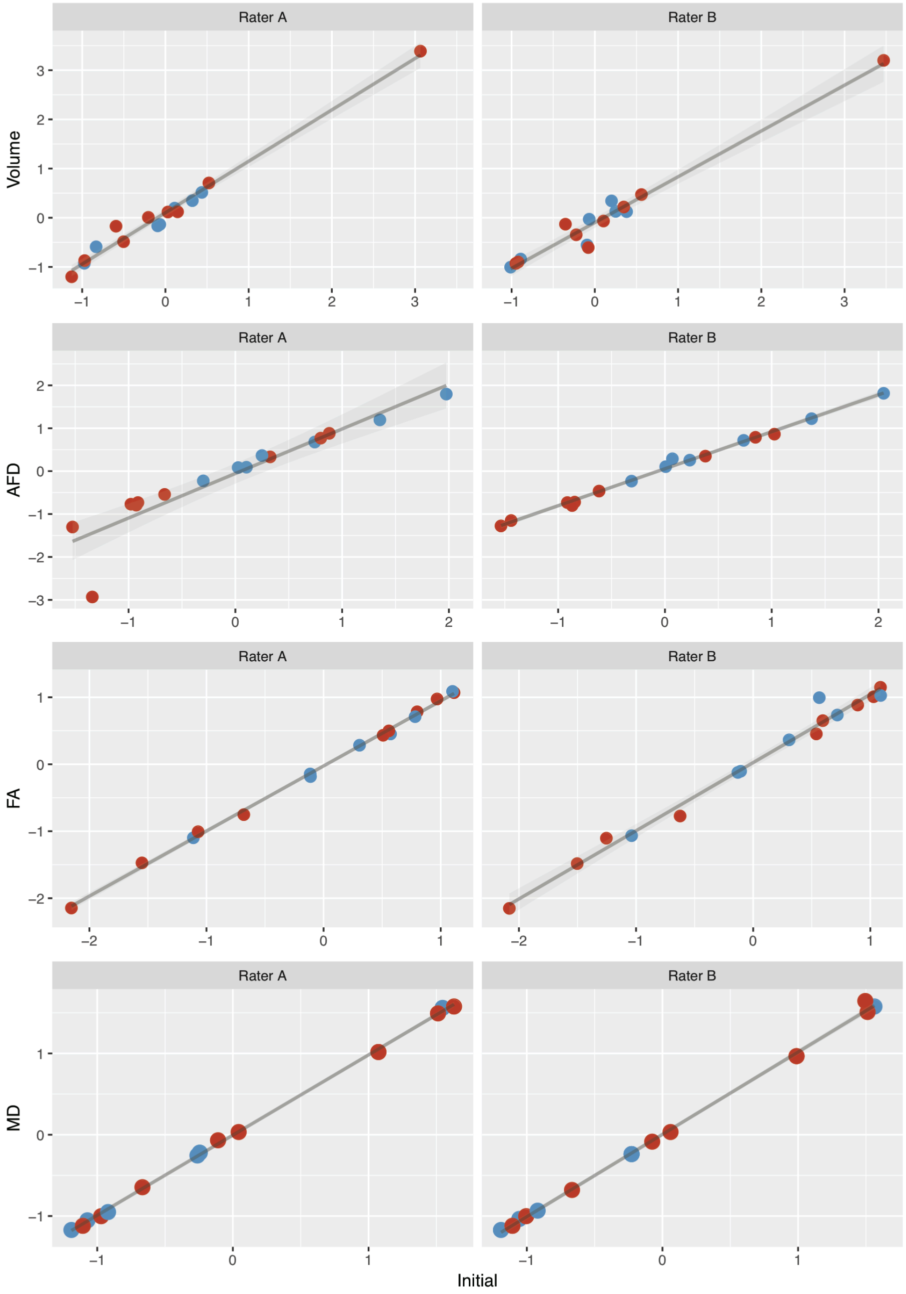

Figure S3. The intra-rater reliability and agreement performance of optic radiation (OR) diffusion metrics and tract volumes. Left column: rater A; right column: rater B. The metrics derived from initial tracking is shown on the x-axis, with repeated tracking shown on the y-axis. Values represent *z*-scores. Red points = metrics derived from the lesional OR, blue points = metrics derived from the non-lesional OR. Volume = tract volume. Abbreviations: AFD = apparent fiber density; FA = fractional anisotropy; MD = mean diffusivity.

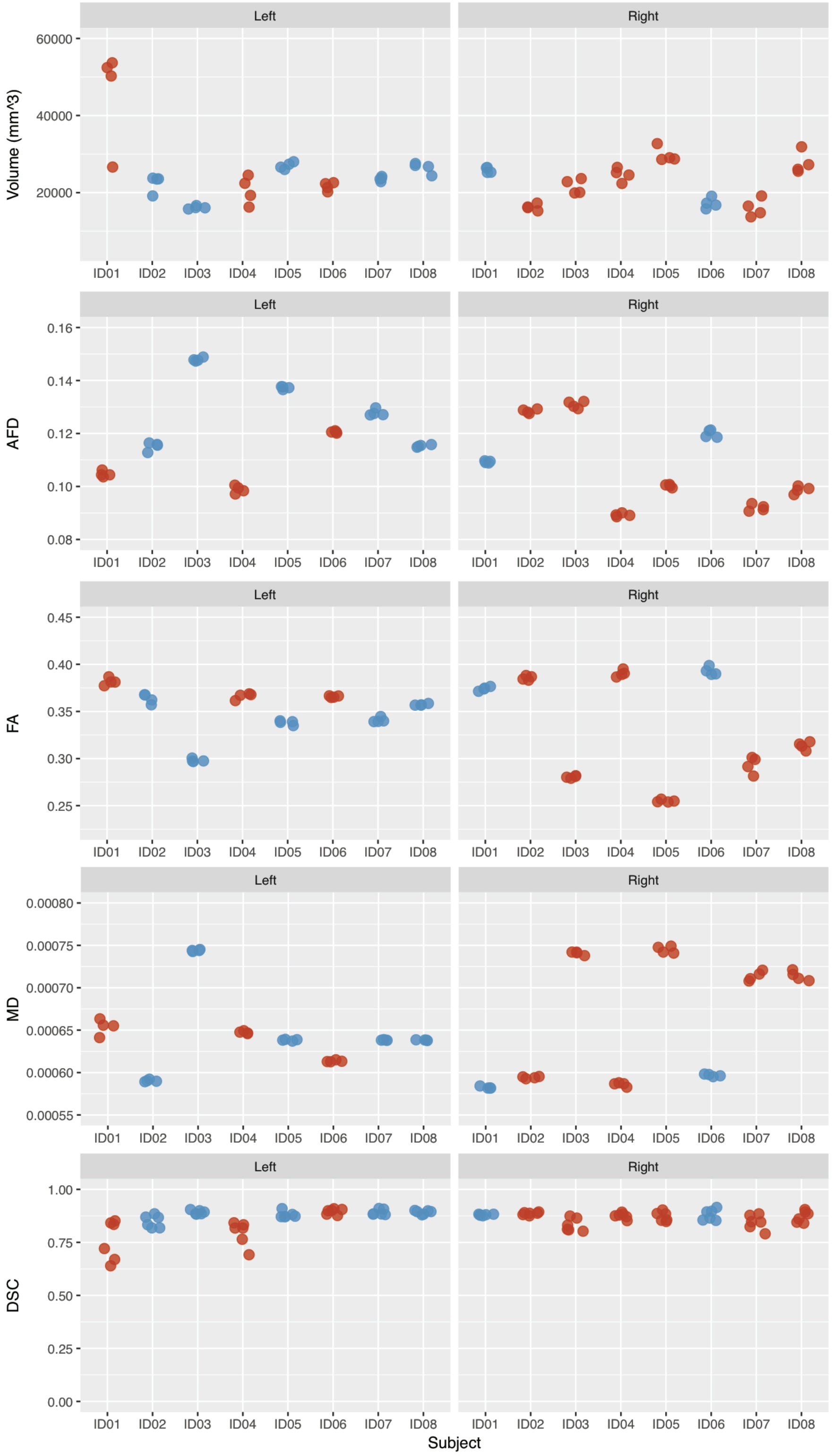

Figure S4. The inter-rater reliability and agreement performance from all raters derived optic radiation (OR) diffusion metrics and tract volumes. The patients are represented on the x-axis, with each point representing a different rater. Red points = metrics derived from the lesional OR, blue points = metrics derived

from the non-lesional OR. Volume = tract volume. Abbreviations: AFD = apparent fiber density; FA = fractional anisotropy; MD = mean diffusivity.
